# Ionic Liquids as Environmentally Benign Electrolytes for High‐Performance Supercapacitors

**DOI:** 10.1002/gch2.201800023

**Published:** 2018-10-23

**Authors:** Suniya Shahzad, Afzal Shah, Elaheh Kowsari, Faiza Jan Iftikhar, Anum Nawab, Benoit Piro, Mohammad Salim Akhter, Usman Ali Rana, Yongjin Zou

**Affiliations:** ^1^ Department of Chemistry Quaid‐i‐Azam University 45320 Islamabad Pakistan; ^2^ Department of Physical and Environmental Sciences University of Toronto Scarborough Toronto M1C 1A4 Canada; ^3^ Department of Chemistry Amirkabir University of Technology Tehran 159163‐4311 Iran; ^4^ Univ. Paris Diderot Sorbonne Paris Cité ITODYS UMR 7086 CNRS, 15 rue J‐A de Baïf 75205 Paris Cedex 13 France; ^5^ Department of Chemistry College of Science University of Bahrain Sakhir 32038 Bahrain; ^6^ College of Engineering King Saud University PO‐BOX 800 Riyadh 11421 Kingdom of Saudi Arabia; ^7^ Guangxi Key Laboratory of Information Materials Guilin University of Electronic Technology Guilin 541004 P. R. China

**Keywords:** electrolytes, energy storage, ionic liquids, supercapacitors, sustainability

## Abstract

Electrochemical capacitors (ECs) are a vital class of electrical energy storage (EES) devices that display the capacity of rapid charging and provide high power density. In the current era, interest in using ionic liquids (ILs) in high‐performance EES devices has grown exponentially, as this novel versatile electrolyte media is associated with high thermal stability, excellent ionic conductivity, and the capability to withstand high voltages without undergoing decomposition. ILs are therefore potentially useful materials for improving the energy/power performances of ECs without compromising on safety, cyclic stability, and power density. The current review article underscores the importance of ILs as sustainable and high‐performance electrolytes for electrochemical capacitors.

## 1 Introduction

The current industrial world is extensively using fossil fuels in order to comply with the ever‐increasing energy demand. However, exhaustion of nonrenewable sources and their major contribution in causing environmental issues demand serious attention toward alternative renewable ways of energy generation for realizing the dream of sustainable zero carbon‐based energy economy.^1^, ^2^ Endeavors for achieving this objective has led to the utilization of hydrogen, tidal, wind, and solar energy sources due to their potential of replacing the current fossil fuels by environmentally friendly renewable energy sources. However, many of these sources are sporadic. Hence, for ensuring constant supply of energy, it is imperative to search for proficient, secure, and sustainable electrical energy storage devices (EES).^3^ In this connection, supercapacitors (SCs) using environmentally friendly ionic liquids (ILs) are the best options as clean energy storage devices. For sustained performance of SCs and addressing their limitations/challenges related to low operating voltage and instability of charge–discharge cycle, it is mandatory to replace the conventional aqueous solvents by ecologically benign ILs.^4^, ^5^, ^6^

ILs are potentially useful electrolytes for improving the energy/power performance of SCs. These liquids are often labelled “tailorable” solvents, as their physical and chemical properties can be tailored for specific applications. However, all features of high thermal stability, safety, and high ionic conductivity may not be achieved in a pure IL. One useful approach in this regard is to decrease the viscosity and reduce contribution of different sources toward resistance of specific capacitance *C*_sp_ by the addition of polar organic solvents into ILs which help in developing strong interactions with the ions present in an IL mixture of organic solvents. Protic and aprotic ILs, IL mixtures with molecular solvents, and IL‐based deep eutectic solvents can be employed as substitutes for the standard aqueous solvents with guaranteed results for future environmentally friendly high performance SCs. In fact, many recent reports focused on unravelling the basic mechanism of ion transfer in bulk electrolytes and interpretation of in situ processes happening at the interface of electrode–electrolyte suggest a switch from basic laboratory experiments of charging/discharging toward the best practical utilization of ILs‐based SCs.

It is worth mentioning here that for energy systems, commonly used nonaqueous electrolytes are made up of solution of salts of ions in organic solvents which are mostly volatile.^7^, ^8^, ^9^, ^10^, ^11^, ^12^ Besides this, charging the system at high rates may result in electric sparks; consequently, electrolytes which are highly flammable may cause the device to explode. Moreover, serious environmental issues may emerge due to release of poisonous electrolytes when the cell is pleated. Therefore, these electrolytes imperil the safe utility of electrochemical capacitors (EC) devices.^13^, ^14^, ^15^, ^16^, ^17^, ^18^ The aqueous solvents on the other hand have limited electrochemical window. Therefore, it is contended to substitute standard aqueous solvents with IL‐based solvents for achieving enhanced energy density (ED) and stability in supercapacitors as a result of their high ionic conductivity, broad EC window, and ecofriendly character.^19^ To put this into perspective, ILs have given new dimensions to the next generation EES devices, as these versatile electrolyte media have the potential of realizing the possible commercialization of SCs as environmental friendly energy tools.^20^, ^21^, ^22^, ^23^ Hence, this substitution with ILs is also highly desirable in the context of green technology which is the need of the present era due to environmental concerns.^24^

For the sake of clarity, **Table** **1** below presents the full list of abbreviations used in this review.

**Table 1:** List of abbreviations used in this review

Abbreviation	Full name
AIL	Aprotic ionic liquid
Azp	Azepanium
BIL	Biredox ionic liquid
BMIm	1‐Butyl‐3‐methylimidazolium
BMPy	1‐Butyl‐3‐methylpyridinium
*C*_sp_	Specific capacitance
DCA	Dicyanamide anion
DES	Deep eutectic solvent
EAN	Ethylammonium nitrate
EC	Electrochemical
ED	Energy density
EDLC	Electrochemical double layer capacitors
EES	Electrical energy storage
EMIm	1‐Ethyl‐3‐methylimidazolium
ESW	Electrochemical stability window
HOPMIm	1‐Hydroxy‐1‐propyl‐3‐methylimidazolium
IL	Ionic liquid
Im	Imidazolium
MIm	Methylimidazolium
MPPip	1‐Methyl‐1‐propyl piperidinium
MPPyr	1‐Methyl‐1‐propyl pyrrolidinium
NMPyr	N‐methylpyrrolidinium
OMS	Mesilate
P_4_VPh	Poly‐4‐vinylphenol
pDADMA	Poly(diallyldimethylammonium)
PIL	Protic ionic liquid
POM	Polyoxometalate
*P*_s_	Power density
Pyr	Pyrrolidinium
RTIL	Room temperature ionic liquid
SC	Supercapacitor
TEMPO	Tetramethylpiperidinyl‐1‐oxyl
Tf_2_N	Bis(trifluoromethylsulfonyl)imide
TFSI	Bis(trifluoromethane)sulfonimide
TMAO	Tetramethylammonium oxalate
TMPA	N‐trimethyl‐N‐propyl‐ammonium
VOC	Volatile organic compounds

## 2 Types of ECs and Preference of ILs Over Traditional Electrolytes

**Figure 1:**
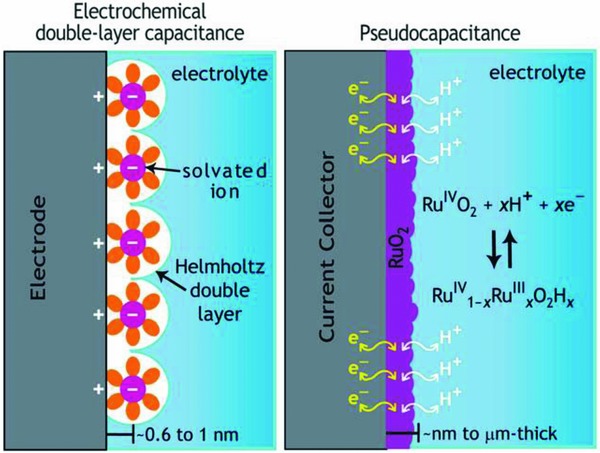
Schematic representation of mechanism of storing charge by two different ECs: EDLC and pseudocapacitance (example of RuO_2_). Reproduced with permission.^32^ Copyright 2011, Cambridge University Press.

ECs can be further divided into electrochemical double layer capacitors (EDLCs) and pseudocapacitors (**Figure** **1**). The fundamental mechanism of storage of charge in EDLCs is based on ion adsorption from the electrolyte on relatively sizeable surface area electrodes that are commonly porous carbon‐based materials. EDLCs have notable power density (*P*_s_), long life span, and quick charge/discharge rate,^25^, ^26^, ^27^, ^28^, ^29^ while pseudocapacitor materials can store even greater amount of charge than EDLCs because of fast and reversible electron transfer reactions taking place at or in the vicinity of the surface of the electrode.^3^ However, this increase in ED and specific capacitance (*C*_sp_) occurs at the cost of cycle life and power density (*P*_s_) relative to EDLCs.^25^, ^30^ When it comes to sustained performance of EC devices and their environmental impact, the pseudocapacitive materials have their own challenges and limitations.^25^ The volumetric or compositional changes that occur in pseudocapacitive materials during charge/discharge cycling can affect the electrode material, which eventually leads to performance degradation over long periods of device operation. Hence, further research activities are underway to address these challenges without compromising the cyclic stability and safety.^31^

Electrolytes play a significant function in regulating the specific capacity, cycle stability, rate, and safety of the EES devices. An electrolyte can reduce or eliminate the probability of parasitic side reactions; hence, the choice of an appropriate electrolyte is vital for getting maximum output. Electrolytes of wide‐ranging electrochemical window and good ionic conductivity are reported to pronouncedly improve the net performance of EES devices.^30^, ^33^, ^34^

**Scheme 1:**
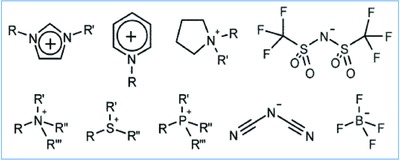
Representative cations and anions utilized to form ILs. Reproduced with permission.^23^ Copyright 2009, The Royal Society of Chemistry.

Structural design of ILs plays a decisive role in controlling their physicochemical properties, for example, their self‐assembly in the electrical double layer. It is largely directed by the type and nature of the constituent cations and anions and their side‐chains.^23^ One advantage of ILs is that the cationic and anionic components, some of which are displayed in **Scheme** **1**, can be selected independently in order to tailor such physicochemical properties.^23^, ^35^ The stability of ILs also depends on their structure. On one side, the presence of delocalized charge centers reduces the possibility of intramolecular interactions.^19^ On the other side, ions of ILs having no or low symmetry limit their packing into a crystal structure of low melting points.^36^, ^37^, ^38^, ^39^

ILs are mostly made up of organic bulky cations (e.g., quaternary ammonium NR_4_^+^, pyrrolidinium Pyr, imidazolium Im, etc.,) and inorganic or organic charged localized anions (e.g., triflate also known as trifluoromethanesulfonate CF_3_SO_3_^−^, hexafluorophosphate PF_6_^−^, halides X, tetrafluoroborate BF_4_^−^, dicyanamide DCA^−^, etc.). The insignificant vapor pressure of these substances is a result of the strong interaction among their constituent ions,^40^ thus their atmospheric volatile organic compounds' (VOCs) contribution is null.^41^ The nonflammability, no or low toxicity, and high mechanical, thermal, and electrochemical stability of these species are also due to these strong coulombic interactions between their constituents.^40^ Furthermore, the most crucial ecologically green aspects of ILs are their recyclability and biodegradability. To further enhance the chances for the discovery of green ILs, efforts are made to derive anions and cations from food grade or natural precursors.^42^ To assist in the formation of less toxic and more ecofriendly room temperature ILs (RTILs), some guidelines framed in 2014 are shown in **Table** **2**.^43^

**Table 2:** Guidelines for more biodegradable and less toxic RTILs formation. Reproduced with permission.^43^ Copyright 2014, Elsevier

IL component	Toxicity		Biodegradability	
	Avoid	Use	Avoid	Use
Anion	Fluorine	Alkyl sulfates, benzenesulfonates, carboxylates	Fluorine	Alkyl sulfates, benzenesulfonates, carboxylates
Cation core	Imidazolium	Morpholinium and pyridinium	Imidazolium	Pyridinium
Substituent	Long hydrophobic alkyl chains	Short polar chains	Polar functional groups	Long hydrophobic alkyl chains

Ionic liquids generally present an electrochemical stability window (ESW) of up to 4–6 V due to poor susceptibility of their cations and anions toward redox processes. Nevertheless, the ESW can further be limited by a component of an electrolyte such as a polymer, or an electrolyte additive, which too can undergo electrochemical reactions at the electrode. **Table** **3** presents the boundaries for ESW of various ILs at cathodic and anodic electrodes.^44^

**Table 3:** ESW of ILs at a glassy carbon electrode using 0.01 m Ag/Ag^+^ in DMSO as the reference electrode. Reproduced with permission.^44^ Copyright 2003, Elsevier

Ionic liquid	Cathodic boundary [V]	Anodic boundary [V]
[EMIm][BF_4_]	−2.1	2.1
[BMIm][BF_4_]	−2.1	2.1
[BMIm][PF_6_]	−2.1	2.0
[EMIm][Tf_2_N]	−2.1	2.0
[BMPy][Tf_2_N]	−1.0	2.0

A downside of ILs is however their comparatively lower electrical conductivity than aqueous systems—an outcome of their high viscosity.^45^ Nevertheless owing to their better thermal stability, electrolytes based on ILs can be used at higher temperatures, thus to some extent reducing this problem.^46^, ^47^

The aforesaid expedient physical and chemical properties render ILs as interesting contenders to improve the energy/power performances of ECs. At the same time, the sensitivity of the ESW of ILs to impurities has been reported by comparing halides and molecular anions containing halides. The oxidation of halides was found to occur much earlier as compared to molecular anions (e.g., stable anions containing fluorine such as bis(trifluoromethylsulfonyl)imide TFSI or [N(Tf_2_)]), which retain a negative charge delocalization over the entire volume. As a result, electrochemical stabilities are much lowered by the presence of halide ions.^40^ There are some ILs whose anions consist of halogens (e.g., [CF_3_SO_3_]^−^, [PF_6_]^−^, [AlCl_4_]^−^, [(CF_3_SO_2_)_2_N]^−^, or [BF_4_]^−^); due to the poor hydrolysis stability of some anions like [PF_6_]^−^ and [AlCl_4_] or presence of water or halides or amines that are left untreated as a consequence of synthesis of ILs, it takes a toll on the “greenness” of the material. For these reasons, extra efforts are required for designing greener ionic liquids.^41^

ILs have added a new dimension in solution chemistry. Due to their characteristic ionic nature, ILs provide, for redox species, an environment which is significantly different from conventional aqueous and nonaqueous electrolytes. Consequently, the free energy of reactants and products is affected, leading to an observable shift in overall redox potentials. Redox reactions which encompass solid species as reactants or products are likely to be affected by the solvent environment. Therefore, it is imperative to probe the probable potential differences of redox reactions that are otherwise well agreed upon in other solvents with regard to ILs. A combined experimental and theoretical work may provide useful information in this regard. On the basis of simulation analysis, Lynden–Bell^48^, ^49^ concluded that the Marcus theory was well applicable to ILs investigated with small cations, whereas the mechanism became complex with large cations where thermodynamic parameters of the solution, e.g., reorganization and activation energies, showed close similarity to those calculated for CH_3_CN. This was due to efficient screening effect of charge due to long range interactions by both categories of solvents, whereas short range interactions became important for molecular dynamics of the solvents that control the orientation of the solvent. A comparative study of molecular solvents versus ILs was made by Zhang et al.,^50^ after studying experimentally the redox behavior of the polyoxometallate species α‐S_2_W_18_O_62_^4−/5−^, it revealed a very strong dependency on the polarity and purity of the solvent, which became more evident with increase in charge on the species.^51^, ^52^, ^53^ As a logical consequence, recently, ILs have been developed by purposefully introducing redox functionality. Such redox ILs are now attracting significant attention as solvent free electrolytes in SCs^54^ and other electrochemical devices.^55^, ^56^, ^57^

ILs are categorized into PIL (protic ionic liquids) and AIL (aprotic ionic liquids). PILs are formed through H^+^ transfer from a Brönsted acid to a base. Furthermore, PILs comprising an available dissociable proton on the protonated organic cation render them as remarkably useful source of proton^58^ for pseudocapacitors which require insertion of H^+^ into the electrode material during the process of charge storage.^59^, ^60^, ^61^ AILs on the other hand, based on alkylimidazolium, phosphonium, and aliphatic quaternary ammonium salts, have been widely studied for use in EDLCs.^44^, ^62^, ^63^, ^64^ The use of AILs is known since long time and they have been extensively used in advanced electrochemical devices like dye sensitized solar cells,^65^, ^66^ batteries,^67^ electrochromics,^68^ and recently in SCs.^69^ By contrast, PILs came on the scene relatively late and have been fetching substantial attention from research groups that seek acid free proton activity in solvents.^70^ Besides this, PILs have also been found useful for thermochemical cells^71^ and SCs.^72^, ^73^ Both AILs and PILs are investigated for improving the electrochemical voltage window in EC devices.^74^, ^75^, ^76^, ^77^, ^78^, ^79^, ^80^, ^81^ In comparison to AILs, the fluidity and conductivity of PILs are usually higher while their melting points are lower. Also, as no byproduct is formed during their synthesis, preparation of such ILs is more convenient, greener, and cheaper. However, according to several reports, the PILs ionicity can be limited in comparison to AILs due to their ability to form a network of hydrogen bonds as in ethylammonium nitrate.^82^

Lately, another emerging type of IL‐based solvents, deep eutectic solvents (DESs) that are inexpensive and eco‐friendly, have attracted great interest from researchers.^83^, ^84^, ^85^, ^86^, ^87^, ^88^ DESs are mainly prepared from food and/or biosources which make them potentially cheaper, less toxic, and more biodegradable as compared to most of the RTILs.^42^ DESs are easy to synthesize and present effective applications for EDLC devices and Li batteries. Based on earlier reports on PILs and solids (plastic crystals), the NH_2_ and OH moieties are believed to be accessible to hydrogen‐bonded donor molecules through Van der Waal interactions, offering promising pathways for fast transfer of proton and paving the way for proton active PIL‐based deep eutectic electrolytes, which can play an active part in the improvement of the capacitance in pseudocapacitors.^62^, ^89^

In this review, several discriminating aprotic and protic ILs, IL mixtures (comprising molecular solvents), and IL‐based DES as well as redox ILs are discussed in the perspective of increased voltage stability of electrolytes, and increased capacitance for electrode materials, for the purpose of high power density EC devices, in an environmentally friendly way.^62^, ^90^

## 3 ILs and the Influence of Their Viscosity on the Performance of SCs

The specific capacitance is highly reliant on the type of electrode material and is enhanced by selecting for example conducting materials like carbon‐based nanomaterials of high surface area in EDLCs. However, electrolytes also bring an important contribution to the capacitance and must be selected discreetly and evaluated to optimize the functional voltage.^3^ When ILs are involved, the performances of EES are also greatly affected by the size and type of cations and anions which tailor the physical and chemical properties of these ILs,^91^ particularly in terms of electrolyte viscosity, closely connected to its conductivity, as well as to the interfacial capacitance. The most common IL families that have been appraised so far as prospective electrolytes for SCs are based on aprotic pyrrolidinium and imidazolium‐based cations as well as quaternary ammonia. Usually, pyrrolidinium‐based ILs exhibit enhanced stability against reduction and oxidation than the ones based on imidazolium and therefore have a high ESW which renders high energy density ED and high voltages for the device. Conversely, the imidazolium‐based ILs generally present lower viscosities, so higher conductivities as compared to the former, which are desirable for fast charge–discharge cycles. Hence, these different features of ILs based on different cations have a significant influence on the *P*_s_ as well as the ED of SCs.^45^ It was also observed that ammonia‐based ILs display a larger ESW as compared to pyridinium‐ and imidazolium‐based ILs, the two latter offering higher cell capacitance, however.^92^ Nevertheless, pyrrolidinium, imidazolium, as well as pyridinium cations have been plagued with toxicity issues affecting the greenness of such ILs as against organic electrolytes used for the same purpose. They have been studied by Hernández‐ Fernández et al.,^93^ who found that pyrrolidinium cations were less toxic than pyridinium and imidazolium ones. As per microtox toxicity analysis, the toxicity of ionic liquids [BMPyr][Cl], [BMPyr][TfO], [HOPMIm][glycolate], and [HOPMIm][FCH_2_COO] was less as compared to commonly used organic solvents like toluene or chloroform. It is identified that the toxicity of IL can be lowered by insertion of a OH^−^ group in the cation's alkyl chain thus resulting in greener ILs.

The choice of the anion is also important. It has been shown that BF_4_^−^ or PF_6_^−^ offer higher capacitances than other anions, due to their sizes which fit to the pore size of the mesoporous carbon electrodes commonly employed in SCs.^19^ RTILs are very viscous relative to organic solvents and aqueous media. However, control on their viscosity and thus conductivity can be implemented by both the counterion and the substituents on the organic ion.^94^ Up to some extent, the viscosity problem can also be solved by designing ILs with proper structural framework thus improving the net performance of the device. It was found that ether appendages in the alkyl side‐chains of ILs can induce considerable flexibility thus reducing the viscosity and enhancing the conductivity, as a result of which the performance of the SCs can be improved.^95^ Similarly, fluorohydrogenated anions were explored for their favorable ionic conductivities due to their low viscosities.^96^ Further tailoring of ILs can also be accomplished through introduction of fluorine‐free dicyanamide anions [N(CN)_2_]^−^.^97^, ^98^, ^99^, ^100^

As discussed, one of the main obstructions for application of ionic liquids is to fabricate ILs of low viscosity and thus high ionic conductance (in the range 10^−3^–10^−1^ S cm^−1^) over a broad range of operational temperature (−30–60 °C), and high thermal stability for safety concern. In many cases, such properties are not obtained for a pure IL, without addition of an organic solvent, e.g., acetonitrile, propylene carbonate, or a range of gamma butyrolactone, mononitriles, amides, and adiponitriles.^73^, ^101^, ^102^, ^103^, ^104^, ^105^, ^106^ Such solvents enhance charge transport, thus the capacitance of the electrolyte is improved compared to pure ILs. Even though current advances in IL‐based EDLCs have been humungous, their complete exploitation entails additional comprehensive understanding of the mechanisms of ion transfer in bulk electrolyte as well as of the processes occurring at the electrode/electrolyte interface.^107^, ^108^, ^109^ ILs of wide ESW and reasonable conductivity above room temperature are under study for high‐voltage SCs to ensure ionic conductivity without being limited by high viscosity, working as “solvent‐free” electrolytes. Polarizability of these ILs likewise directly affects the electrode/electrolyte interface and, hence, capacitance of SCs.^34^

As discussed above, viscosity and series resistance are reduced by appending polar organic moieties onto the nitrogen of the pyrrole ring, in pyrrolidinium‐based ILs.^33^ The polarity of such additives helps at lowering the intermolecular interactions, decreasing the viscosity, and therefore improving the conductivity. Applying this strategy, Zhang et al.^110^ have investigated a MnO_2_‐based supercapacitor that uses an electrolytic mixture of [BMIm][PF_6_] and *N,N*‐dimethylformamide (DMF). The authors demonstrated a high operating voltage (≈3 V) and a high specific energy of 67.5 W h kg^−1^ at a fixed power of 593.8 W kg^−1^. As inferred by authors, the addition of DMF in [BMIm][PF_6_] radically increased the diffusion of electrolyte in MnO_2_ (acting as pseudocapacitive material), and in doing so increased the capacitance. It is also significant to consider the contribution of bulky ions of ILs on the pseudocapacitance, for example, on MnO_2_ electrodes, as investigated through in situ X‐ray absorption spectroscopy by Lee et al.^111^ This work indicates that thiocyanate (SCN^−^) anions from [EMIm][SCN] IL can reversibly be intercalated or expelled to and from the tunnels located in octahedral subunits of manganese dioxide.

The most acknowledged ILs hitherto include those which contain weak Lewis bases such as BF_4_^−^, CF_3_SO_3_^−^, Tf_2_N/TFSI^−^, and PF_6_^−^. The literature indicates that transition metals are usually insoluble in the corresponding ILs. On the contrary, DCA anion, being smaller in size than, e.g., bis(trifluoromethane)sulfonimide (TSFI) with the charge on the imide nitrogen delocalized on the whole molecule, is known for facilitating ion–ion interactions. Compared to TSFI, DCA‐based ILs exhibit lower viscosity which leads to a more effective mass transport and an increased conductivity.^112^

## 4 ILs for EDLCs and Hybrid SCs

### 4.1 ILs Based on Imidazolium as SC Electrolytes

#### 4.1.1 Graphene‐Based Electrodes

**Figure 2:**
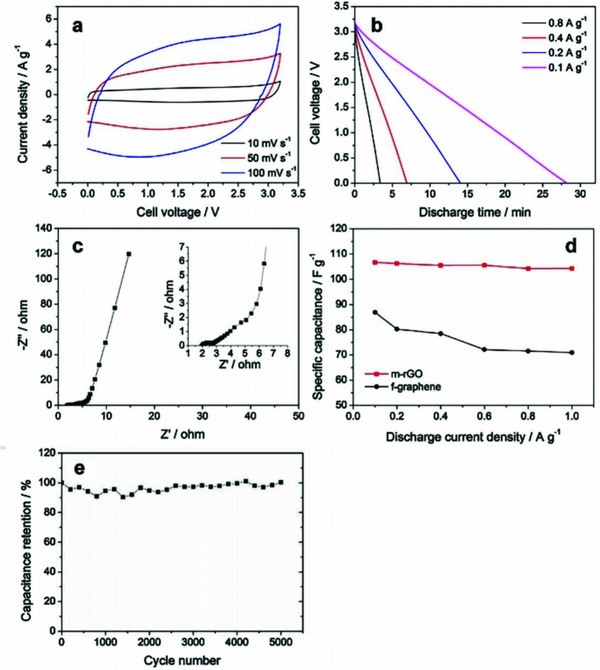
Capacitive properties of m‐rGO supercapacitor in [EMIm][Tf_2_N] IL electrolyte. a) CV at different scan rates; b) galvanostatic discharge curves; c) Nyquist plot (inset shows high‐frequency section); d) capacitance retention at different discharge current densities (m‐rGO capacitance is denoted by a red line while f‐graphene capacitance is denoted by a black line); e) retention of capacitance of the m‐rGO supercapacitor under galvanostatic charge/discharge cycles at 0.5 A g^−1^. Reproduced with permission.^113^ Copyright 2017, Elsevier.

Jeon et al. investigated mesoporous reduced graphene oxide (m‐rGO) as an electrode for SC in [EMIm][Tf_2_N] electrolyte. The capacitive properties of such a SC, *C*_sp_ of 104.3 F g^−1^ at 1 A g^−1^ and a 3% reduction of capacitance after 5000 cycles, are shown in **Figure** **2**.^113^

In order to improve the compatibility between the electrolyte and the electrode, Shao et al.^114^ used [EMIm][BF_4_] as electrolyte but also grafted this IL on the graphene‐modified electrode surface, improving the compatibility between the electrolyte and the electrode. Li et al.^115^ have investigated the interactions between graphene‐modified electrodes bearing oxygen functional groups and three kinds of imidazolium‐ and piperidinium‐based IL electrolytes, namely, [EMIm][BF_4_], [EMIm][Tf_2_N], and [MPPip][TFSI]. In their report, the highest ED (169 Wh kg^−1^ charged to 4.4 V) was attained by using [MPPip][TFSI]. Bag et al.^116^ describes a strong and persuasive rationale to covalently functionalize rGO with ILs based on hydrophilic imidazolium (Im‐IL), for preventing restacking of the adjacent layers for increased wettability and enhanced capacitance. The device could deliver 2 kW kg^−1^ of *P*_s_ and 36.7 Wh kg^−1^ of ED with good cycling stability.

It was demonstrated that the preparation of highly porous graphene‐derived carbon with high porosity and hierarchical structures is highly desirable for improvement of ion diffusion into the pores.^117^ These as‐produced graphene‐based carbons displayed a gravimetric *C*_sp_ of 174 F g^−1^ and a volumetric one of ≈100 F cm^−3^ with a [EMIm][Tf_2_N]/CH_3_CN mixture as electrolyte. Tamilarasan and Ramaprabhu^118^ conducted a systematic study on a SC electrode material based on nitrogen‐doped hydrogen exfoliated graphene and [BMIm][Tf_2_N] as electrolyte. The supercapacitor showed a *C*_sp_ of 170.1 F g^−1^ compared to that with unmodified graphene (124.5 F g^−1^), both obtained at a specific current density of 2 A g^−1^. The device showed a charge storage capacity of 72.4 Wh kg^−1^, an operating voltage of 3.5 V, and an improved cyclic stability. Li and co‐workers^119^ have worked on a high‐performance SC material based on a modified 3D porous graphene‐based material synthesized by mild hydrothermal treatment of graphene oxide (GO) using dual spacers such as [BMIm][Cl] and SiO_2_ spheres so that the interspace between the graphene sheets is enlarged and restacking is avoided. This yielded a maximum *P*_s_ of 13.3 kW kg^−1^ and an ED of 7.0 W h kg^−1^ with almost no loss after 3000 cycles (electrolyte: 1 m H_2_SO_4_). Sasi et al.^120^ have reported preparation of an imidazolium‐based ionic liquid crystal from 3‐pentadecylphenol derived from cashew nutshell. They found that this composition facilitated the crystalline ordering, increasing the ionic conductivity, and displayed equally excellent capacitive properties by engaging symmetrical SCs using mesoporous carbon electrodes and the IL in question as electrolyte. They found a *C*_sp_ of 131.4 F g^−1^ at a current density of 0.37 A g^−1^ and 80% retention of capacitance after 2000 cycles.

#### 4.1.2 Electrodes Based on Other Carbon Materials

EDLC capacitor based on microporous glucose‐derived activated carbon (GDAC) in [EMIm][BF_4_] was chosen by Tooming et al.^121^ for its high bulk conductivity (κ = 13.6 mS cm^−1^) and electrochemical stability within a wide potential region. The *C*_sp_ of 158 F g^−1^ and other electrochemical characteristics like charge and discharge curves suggested that the GDAC‐based SC was competitive.

Outstanding electrochemical properties in the form of enhanced specific capacitance were also achieved for carbon/carbon EDLC using a mixture of [BMIm][OMS] and a polymer electrolyte poly(vinylalcohol) ammonium acetate (PVA–CH_3_COONH_4_) in a cell reported by Liew et al., where the IL increases the conductivity of the electrolyte;^122^ a *P*_S_ of 18.4 kW kg^−1^ and an ED of 0.17 Wh kg^−1^ were observed at ambient temperatures. In the recent years, imidazolium‐cation based ILs like [EMIm][DCA] and [EMIm][SCN] have been reported with conductivities above 20 mS cm^−1^.^40^ In another study by Liew et al., proton conducting polymer electrolytes were synthesized by using CH_3_COONH_4_/[BMIm][Br] through solution casting, for carbon‐based EDLC electrodes.^123^ The *C*_sp_ was 21.89 F g^−1^. Lyu et al.^124^ reported a carbon nanotube/stainless steel two‐ply yarn‐based SC electrodes coated with a [EMIm][BF_4_] gel electrolyte. The ESW of such SC was improved, from 1.0 to 2.7 V. Such an SC presented excellent electrochemical performances with an ED of 6.67 × 10^−2^ Wh cm^−3^ and a high volumetric capacitance of 263.3 F cm^−3^. Hu et al.^125^ demonstrated the use of an aqueous solution of [BMIm][HSO_4_], a protic IL, as a substitute for H_2_SO_4_ in an asymmetric SC. The positive electrode was made up of a nanoporous carbon electrode into which polyoxometalate ions were confined; polyoxometalate (POM) ions brought an increase in *C*_sp_ at 1 mV sec^−1^ of around 90% compared to a similar electrode without POM.

Cui et al.^126^ have reported a rational design of an electrode material based on porous carbon derived from chestnut shells, using [EMIm][BF_4_], [EMIm][Tf_2_N], and [BMIm][BF_4_] as electrolytes, that results in a significant improvement of the SC's performance. The symmetric SC based on [EMIm][BF_4_] displayed a value of 208.7 F g^−1^ as highest *C*_sp_ at a current density of 0.1 A g^−1^ in comparison to similar SCs based on [EMIm][Tf_2_N] and [BMIm][BF_4_] having *C*_sp_ of 180.8 and 34.6 F g^−1^, respectively. After 2000 galvanostatic charge–discharge cycles, their SCs retained 62.9%, 94.5%, and 71.5% of their initial capacitance, respectively. This highlights the electrochemical stability of SCs assembled with [EMIm][Tf_2_N].

Jang et al.^127^ reported a solid/gel polymer electrolyte‐based SC by employing an activated carbon electrode functionalized by a [EMIm][BF_4_]‐containing PVA/H_3_PO_4_ gel. Adding 50% w/w of IL was shown to increase the *C*_sp_ to 271 F g^−1^ at a discharge current of 0.5 A g^−1^, compared to a pristine PVA/H_3_PO_4_‐based SC for which *C*_sp_ = 103 F g^−1^ under the same discharge conditions. *P*_s_ and ED were also enhanced to 23.9 kW kg^−1^ and 54.3 W h kg^−1^, respectively. In another example, high‐energy density SCs based on mesoporous carbons (MCs) were synthesized by He et al.,^128^ from husk of rice by a one‐step ZnCl_2_ activation assisted by microwave. The ED and *C*_sp_ of the electrodes synthesized from a mixture of MC_4_ at a mass ratio of 4:1 for ZnCl_2_:rice husk and MC grew with MC_4_ mass percentage, reached 84 Wh kg^−1^ and 157 F g^−1^ at 0.05 A g^−1^ of current density, respectively. The as‐prepared MC electrode in [BMIm][PF_6_] displayed good cyclic stability in comparison to conventional aqueous and organic electrolytes.

Several solid‐state symmetrical SCs, employing mixtures of 1:3, 1:3.5, 1:4, and 1:4.5 (weight fractions) of cross‐linked poly‐4‐vinylphenol (c‐P_4_VPh):[EMIm][Tf_2_N] as electrolytes have been studied by Ahn et al.^129^ With an optimal ratio of 1:3.5, they found a *C*_sp_ of 172.4 F g^−1^ and an ED of 72.2 W h kg^−1^, for a porous carbon‐based symmetrical SC. They demonstrated a good stability over 1000 cycles in the 0–4 V range. A nanostructured cobalt hydroxide Co(OH)_2_ deposited onto graphene and carbon nanotubes‐containing electrodes was shown to facilitate diffusion of electrolytic ions and was used as electrodes in asymmetric SCs, which displayed a *C*_sp_ of 310 F g^−1^ at 2 A g^−1^ of charging current density, an ED of 172 Wh kg^−1^ and a *P*_s_ of 198 kW kg^−1^ with [EMIm][Tf_2_N] as electrolyte.^130^ In another publication, Chupp et al.^131^ reported chitosan‐based gel electrolytes containing various ratios of adipic acid, acetic acid, [BMIm][BF_4_], and lithium chloride. Ionic conductivities were compared to chitosan/adipic acid and chitosan/acetic acid binary electrolytes. Ewert et al.^132^ have investigated matching compatibility of various porous N‐doped hierarchical carbon materials with different ionic liquids. They have shown the importance to adapt the IL with the electrode material, more particularly in terms of capacitance and long‐term stability; [EMIm][BF_4_] and [EMIm][Tf_2_N] were more particularly investigated.

Francis et al.^133^ have reported fabrication and characterization of a gel electrolyte of poly(vinyl alcohol) + magnesium triflate (Mg(CF_3_SO_3_)_2_) + [BMIm][Br]. The electrochemical potential window of such an ionic electrolyte was reported to extend from 1.35 to 2.6 V, with a significant *C*_sp_ enhancement up to 46 F g^−1^ compared to the IL‐free electrolyte. A very original approach was described by Kim et al.,^134^ who carbonized an imidazolium IL to produce an outstanding nanoporous graphitic material of high surface area, which could be promising for use in supercapacitors. Kim et al.^135^ demonstrated the significance of a finely tuned porous structure with regard to a carbon nanofiber‐based supercapacitor using [EMIm][Tf_2_N] that exhibited a *C*_sp_ of 161 F g^−1^ and a very high ED of 246 Wh kg^−1^. As others, they demonstrated that matching the pore diameter with the size of the IL is critical.

At last, Lian et al.^136^ have investigated the electrical double‐layer structure and capacitance of onion‐like carbon electrodes with two [EMIm][Tf_2_N] and [EMIm][BF_4_]. They corroborated experimental results with DFT calculations and concluded that the mixture of the two ILs makes more co‐ions to leave and more counterions to stay on the electrode surface, leading to an increase of the counterion density within the EDL structure and hence improves capacitance.

#### 4.1.3 Electrodes Based on Other Materials

**Figure 3:**
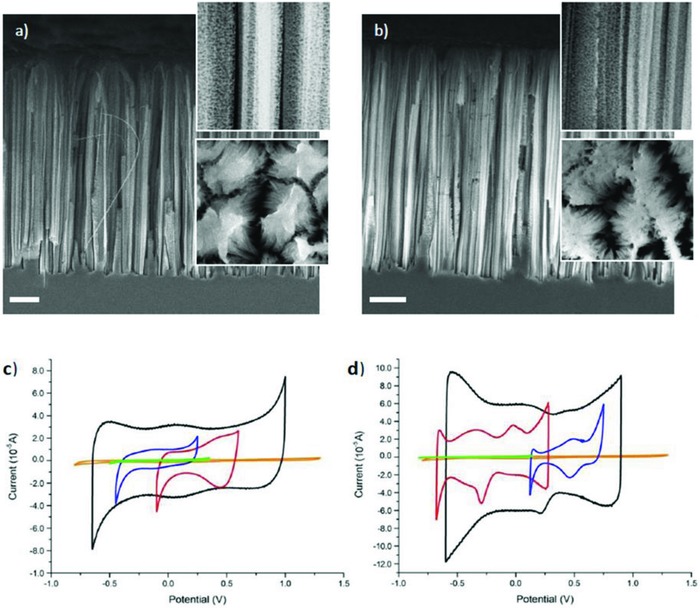
SEM of cross‐sectional area of SiNW: a) not oxidized and b) oxidized. Inset shows top: cross‐sectional zoomed in view; bottom: zoomed‐in view of SiNW bunching. Cyclic voltammograms of 5 working electrodes in [BMIm][Tf_2_N] at a scan rate of 50 mV s^−1^ for HNO_3_‐oxidized SiNW (black), electro‐oxidized SiNW (red), SOD‐treated SiNW (blue), bulk Si (green), and bulk Au (orange) for c) HNO_3_‐oxidized n‐type SiNW at 120 °C and d) HNO_3_‐oxidized p‐type SiNW at 90 °C. Reproduced with permission.^137^ Copyright 2016, Elsevier.

For improving the performance of capacitor electrodes in IL, Qiao et al.^137^ reported the use of [BMIm][Tf_2_N] with n‐type and p‐type Si nanowire (SiNWs) electrodes obtained by metal‐supported chemical etching. To increase the doping density of the SiNWs, they were oxidized in hot concentrated HNO_3_, which led to a tenfold increase in current and *C*_sp_ of 238 µF cm^−2^ (0.4 F g^−1^, 159 mF cm^−3^) and 404 µF cm^−2^ (0.7 F g^−1^, 269 mF cm^−3^) for the n‐type and p‐type SiNWs electrodes, respectively (**Figure** **3**).

### 4.2 Pyrrolidinium‐Based ILs as SC Electrolytes

From a practical point of view, air‐stable ILs were found particularly attractive, for instance [BMPyr][TFSI], [MPPyr][TFSI], [MPPip][FSI], [pDADMA][TFSI], or others.

#### 4.2.1 Boron‐Doped Diamond Electrode

Applications of boron‐doped diamond electrodes for supercapacitors were proposed for the first time by Gao et al.,^138^ by coating silicon nanowires with 100 nm thick layer of nanocrystalline diamond through a bottom‐up template growth method. To increase the potential window up to 4 V, [MPPyr][TFSI] was used as electrolyte. The capacitance of such diamond‐based electrodes reaches 105 µF cm^−2^, boosting up the energy storage capacity to around 50 times as compared to unmodified Si wires. The authors reported a *P*_s_ of 0.94 mW cm^−2^ and an ED of 0.02 µWh cm^−2^, along with a charging/discharging stability over 10 000 cycles.

#### 4.2.2 Carbon Based Electrodes

Compact graphene films, as prepared by Yang et al.,^139^ were used as SC electrodes by Lin et al.^140^ in an eutectic IL mixture comprising a 1:1 weight ratio of [BMPyr][FSI] and [MPPip][FSI]. The temperature window extended from −30 to 80 °C; the ESW was 3.5 V below room temperature. The maximum gravimetric capacitances at 80, −20, and −30 °C were reported to be 175 F g^−1^ (85 mAh g^−1^), 130 F g^−1^ (63 mAh g^−1^), and 100 F g^−1^ (49 mAh g^−1^), respectively. A 60 µm thick graphene film exhibited a 50 F cm^−3^ volumetric capacitance.

Durable, self‐supporting solid ion gel polymer electrolytes, with moduli >100 MPa and presenting outstanding ambient ionic conductivities, i.e., over 10^−3^ S cm^−1^, a capacitance of 2 µF cm^−2^ and a very wide stability window of 5.6 V at RT were prepared by Mantravadi et al.,^141^ by employing [BMPyr][TFSI] with methyl cellulose (MC) as gel former. For that, [BMPyr][TFSI] was first dissolved in a mixture of MC in DMF that was subsequently removed to form a thin, flexible, self‐standing ion gel containing up to 97 wt% IL. Similarly, a SC using a gel electrolyte and activated carbon electrodes was described by Nègre et al. An IL mixture of [BMPyr][FSI] and [MPPip][FSI] was entrapped into a SiO_2_ network, forming an ion gel^142^ used as electrolyte as well as separator, operated over a 3 V window. This solid‐state SC exhibited a *C*_sp_ of up to 95 F g^−1^ at room temperature, due to the filling of the carbon pores by the ions of the gel. Tiruye et al. have reported on the performance of electrolytes made up of a mixture of a conventional polymer based pyrrolidinium IL, [BMPyr][TFSI], with a polymeric IL (IL‐*b*‐PE) consisting of a 40:60 ratio w/w of [pDADMA][TFSI], poly (diallyldimethylammonium) bis (trifluoromethanesulfonyl) imide. Using activated carbon electrodes impregnated with this electrolyte, the resulting symmetric SC showed a *C*_sp_ of 100 F g^−1^ and an ED of 32 Wh kg^−1^. Following another approach, Patil et al.^143^ have reported functionalization of stainless still collectors with polyaniline obtained by electropolymerization of aniline in [NMPyr][HSO_4_]. Under optimized conditions, they obtained a *C*_sp_ of 581 F g^−1^ and an ED of 96.6 Wh kg^−1^ at 1 mA cm^−2^.

Two different pyrrolidinium‐based ILs, [MPPyr][FSI], [BMPyr][TFSI], as well as imidazolium [EMIm][Tf_2_N] were compared by Dagousset et al.,^144^ who observed that binary mixtures of the corresponding ILs with 50 wt% γ‐butyrolactone (GBL) gave interesting properties for SC applications, in a temperature range from −50 to 100 °C. Yu and Chen^145^ reported an interesting concept of “supercapattery,” a hybrid design of an ideal electrode having capacitor‐like and battery‐like features, by utilizing an IL‐based electrolyte containing [BMPyr][FAP], gamma‐butyrolactone (γ‐GBL), and LiClO_4_. The device used a lithium negative electrode and a microporous activated carbon (AC) positive electrode and displayed an energy capacity of up to 230 Wh kg^−1^ at a current density of 1 mA cm^−2^. [BMPyr][TFSI] has also been used along with nanofiber electrodes made of a mixture of carbonized nanocellulose and activated carbon. Such EDLCs displayed efficient electron transport and high capacitance.^146^

#### 4.2.3 Micro‐Supercapacitors

Vertically oriented graphene nanosheet (VGN) electrodes with deposition of highly doped nanostructured Si were employed by Aradilla et al.,^147^ for integrated miniaturized energy storage devices. A symmetric micro‐supercapacitor was assembled from two VGN electrodes separated by [MPPyr][TFSI] as electrolyte in a planar configuration. The device displayed a quasi‐ideal capacitive behavior with a specific capacitance of 2 mF cm^−2^, a *P*_s_ of 4 mW cm^−2^, an ED of 4 µW h cm^−2^, and operated within a large cell voltage of 4 V. An outstanding stability was demonstrated, the device retaining 80% of its initial capacitance after 150 000 charge/discharge cycles at 1 mA cm^−2^. Aradilla et al.^148^ also studied another hybrid 3D micro‐supercapacitor device by coupling polypyrrole as pseudo‐capacitor material and Si “nanotrees” as EDLC material, along with an aprotic ionic liquid (APIL) electrolyte such as [MPPyr][TFSI]. The device showed a high *C*_sp_ of ≈14 mF cm^−2^ and an ED of ≈15 mJ cm^−2^ at a 1.5 V cell voltage. It showed a retention of 70% of its initial capacitance after thousands of charge/discharge cycles at 1 mA cm^−2^. Dubal et al.^149^ reported a novel hierarchical design of heterostructures made of ultrathin MnO_2_ nanoflakes@silicon nanowires (MnO_2_@SiNWs) used as electrode material in a Li‐ion doped [MPPyr][TFSI] IL. This µ‐SC was cycled at a high operating voltage of 2.2 V and gave a capacitance of 13 mF cm^−2^.

### 4.3 ILs based on Ammonium as SC Electrolytes

**Figure 4:**
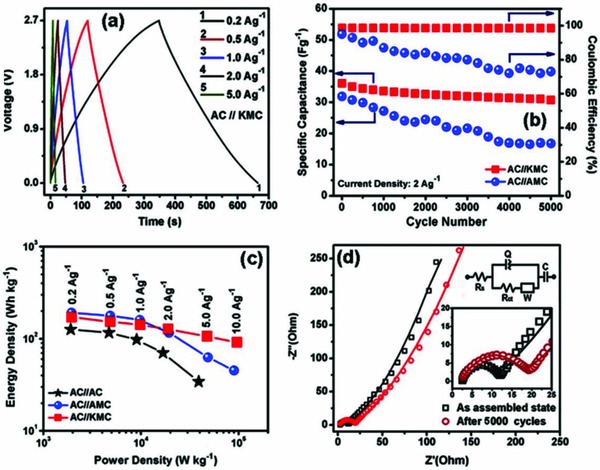
a) Galvanostatic charge–discharge profiles at different current densities (0.2–5.0 A g^−1^) of a AC//Montmorillonite/MWCNT/MnO_2_ cell. b) Stability under cycling of AC//Montmorillonite/MWCNT/MnO_2_ and AC//AC/MWCNT/MnO_2_ cells. c) Ragone plots of AC//Montmorillonite/MWCNT/MnO_2_, AC//AC/MWCNT/MnO_2_, and AC//AC cells. d) Electrochemical impedance spectra for the AC//Montmorillonite/MWCNT/MnO_2_ cell, and equivalent circuit used for fitting. Reproduced with permission.^152^ Copyright 2016, Elsevier.

Perananthan et al.^150^ reported recently the use of tetramethylammonium oxalate (TMAO) in supercapacitor electrodes. TMAO was incorporated in polyacrylonitrile fibers which were carbonized and activated to obtain TMAO/carbon nanofibers. They reported a high specific surface area of 2663 m^2^ g^−1^, comparable to theoretical value for graphene of 2600 m^2^ g^−1^. They found a *C*_sp_ of 140 F g^−1^. At a 1 A g^−1^ of discharge current density, they obtained 68 Wh kg^−1^ and 1.7 kW kg^−1^ of ED and *P*_s_, respectively. The device showed a capacitance loss of only 20% after 1000 cycles. Bettini et al.^151^ used N‐trimethyl‐N‐propyl‐ammonium bis(trifluoromethanesulfonyl) imide [TMPA][TFSI] as electrolyte in a µ‐SC electrode made of carbon deposited by supersonic cluster beam deposition on a Mylar sheet. The µSC were operated at 3 V at 80 °C with a capacitance density of ≈10 F cm^−3^ at a maximum specific power and energy densities of 8–10 W cm^−3^ and 10 mWh cm^−3^, respectively. The authors reported a long‐term stability of over 2 × 10^4^ cycles. Maiti et al.^152^ reported [Et_4_N][BF_4_] (tetraethylammonium tetrafluroborate) in CH_3_CN as electrolyte in a SC based on a novel hybrid composite of multi‐walled carbon nanotubes (MWCNTs), MnO_2_, and montmorillonite. The electrochemical properties of the clay‐based SC were studied in an asymmetric cell against AC. The EDLC SCs were found to operate in a wide electrochemical potential window of 0.0–2.7 V and showed an ED of 171 Wh kg^−1^ and a *P*_s_ of 1.98 kW kg^−1^. The authors attributed the performances to acid–base interactions between the clay and the Et_4_N^+^ cations (**Figure** **4**). In another study, [TEA][TFSI] was evaluated in an EDL SC based on activated charcoal electrodes containing hydroquinone as electron shuttle. Galvanostatic charge–discharge measurements showed an improved *C*_sp_ of 72 F g^−1^ and a specific ED of 31 Wh kg^−1^.^153^

### 4.4 Other ILs as SC Electrolytes

Pohlmann et al.^154^ have introduced a new class of promising, innovative, greener, and cheaper electrolyte for EDLCs as compared to well‐established ILs, namely, azepanium‐based ILs such as Azp_14_TFSI and Azp_16_TFSI, which have been compared with [BMPyr][TFSI]. It has been reported that the large cation size contributes to lowering the conductivity of the electrolyte resulting in no noticeable change in comparison to other ILs; however, these two azepanium ILs showed comparable energy storage capability and cycling stability with up to 3.5 V operative voltage and no noticeable degradation. Romann et al.^155^ validated a SC device working at high voltage, i.e., up to 10 V, using an IL based on DCA and graphene‐based electrodes. They also showed passivation of these electrodes at 10 V, due to anodic polymerization of the [BMPyr][DCA] IL. This process led to formation of a self‐healing protective nanolayer on the electrodes, increasing the specific area and the ED. Huang et al. also reported the use of graphene nanosheets, in various IL electrolytes ([EMIm^+^] and [BMPyr^+^] as cations, [TFSI]^−^, [BF_4_]^−^, and [DCA]^−^ as anions).^156^ [BMPyr][DCA] was shown to be the best electrolyte, giving a *C*_sp_ of 235 F g^−1^ within a potential range of 3.3 V and maximum *P*_s_ and ED of 43.3 kW kg^−1^ and 103 Wh kg^−1^, respectively, much higher than with a conventional organic electrolyte‐based control cell, giving *P*_s_ and ED of 17.6 kW kg^−1^ and 19 Wh kg^−1^, respectively.

## 5 Pseudocapacitors with IL as Electrolyte

Pseudocapacitors offer higher capacitance and are therefore able to store more energy compared to EDLCs of the same surface area. However, pseudocapacitive materials rely on redox reactions like batteries and thus exhibit lower stability upon charge–discharge cycling.^157^, ^158^ However, various pseudocapacitive metal oxides like ruthenium dioxide (RuO_2_) display outstanding capability in terms of power and energy densities in highly protic nonaqueous media, due to coupled electron–proton transfer reactions that take place at the vicinity of the electrode material, which is considered as the origin of the high capacitance of such materials.^61^, ^159^, ^160^ Values of capacitance over 700 F g^−1^ have been known to result from oxygenation and hydrogenation at lower potential in alkaline and acidic electrolytes, respectively.^161^ RuO_2_ reveals high *C*_sp_ values of about 150–260 F cm^−2^ which are approximately ten times greater than that for carbon.^12^ The reaction between H and Ru ions is believed to be the reason for such high capacitance. The current–voltage curve of RuO_2_ obtained in an H_2_SO_4_ electrolyte is mainly without any features and is mirror‐like within a 1.4 V potential range. However, since the up‐and‐coming challenges in pseudocapacitors where H^+^ is produced by a mineral acid in aqueous‐based protic media, safety issues, and corrosion, considering nonaqueous protic media which can circumvent these drawback, is of high interest.^61^ Additionally, using aqueous electrolytes for the purpose of capacitive charge storage limit the potential window for EC devices to ≈1 V; high capacitance values are not possible to achieve for such low operating voltages.^162^ However, a robust possibility is to address all such challenges related to supercapacitors based on metal oxides (e.g., RuO_2_) by replacement of conventional aqueous solvents with ecologically benevolent IL‐based solvents. For example, using nonfluorinated PILs (protic ILs) which are environment‐friendly is of high interest. Binary eutectic composition of PILs based on [EMIm][HSO_4_], [MIm][HSO_4_], or [Im][HSO_4_] incorporated into polymer electrolytes such as poly(ethyleneoxide) has been reported by Ketabi et al.^163^ Rochefort and Pont^61^ have reported prime investigations on PILs application for metal oxide pseudocapacitors. In this review, the authors have reported that RuO_2_ electrodes showed specific capacitance in protic [MPy][TFA] of *C*_s_ = 83 F gm^−1^, of the same order of magnitude than that observed in aqueous solvents where electron–proton transfer reactions are responsible for pseudocapacitance of the material. At the same time, they have also reported that the same RuO_2_ electrodes exhibited exceedingly poor response in an APIL [EMIm][BF_4_]. In a follow up investigation, with purpose of understanding structure–properties associations, Mayrand‐Provencher et al.^164^ have studied pyridinium‐based PILs for their use as electrolyte in pseudo capacitors based on metal oxide electrodes. The authors found that the PILs with 1:2 molar ratio of base:acid showed higher ionic conductivities and low viscosities compared to PILs with 1:1 molar ratio. The authors explored a range of pyridinium based PILs in that study with varying alkyl chain lengths and substituent positions. Upon using these PILs for thermally prepared RuO_2_ pseudocapacitor electrodes, it was found out that the *C*_sp_ depends on scan rate, displaying low *C*_sp_ at high scan rates for PIL having longer alkyl chains and thus larger viscosities. The basis for this observation is the relative long time required for the ions to get access to inner, less accessible, regions of the electrode which is hindered at high scan rates. Thus, in the light of both studies on the ILs application for RuO_2_ SC, it can be deduced that even though ILs have comparative high viscosities and hence low ionic conductance, PILs and their compositions rich in acid or base exhibit important properties to attain high charging rate for energy storage devices.

The scarcity of ruthenium metal and therefore its high cost and toxicity renders the deployment of Ru based materials challenging for their wide spread use. Hence, there is an acute demand of materials that can display high specific capacitance and cyclic stability, while still being economical and easily available for their use in electrochemical charge storage devices.^165^, ^166^ One such promising material that displays appreciable characteristics both for electrocatalysis (such as water splitting) and charge storage (such as electrochemical capacitor) applications is manganese dioxide (MnO_2_).^167^, ^168^, ^169^, ^170^, ^171^ Oxide film preparation by means of electrochemical deposition holds the advantage of controlled conditions.^172^ For example, the film thickness and the oxide composition can easily be adjusted by modifying the charge passed, the precursor ion concentration in the electrolyte, and the applied potential.^173^ Maiti et al.^174^ have accomplished comparative study of nanostructured mesoporous MnO_2_ hollow spheres as electrode material and the influence of electrolyte based on imidazolium on their performance by fabrication of AC/MnO_2_ asymmetric SC cells in ILs based on imidazolium as electrolytes. Four different ILs were investigated electrochemically by combining four different anions [(PF_6_^−^), (BF_4_^−^), (CF_3_SO_3_^−^), and (TFSI^−^)] and two different cations [EMIm^+^] and [BMIm^+^]. Their influences on the physicochemical properties such as ionic size, viscosity, and nucleophilicity were analyzed to develop high performance IL‐based SCs. Similarly, copper oxide (CuO) was investigated by Awale et al., as pseudocapacitor electrode, due to its ecofriendly nature and low cost, along with a novel IL, 1‐(1′‐methyl‐2′‐oxo‐propyl)‐2,3‐dimethylimidazolium chloride, as electrolyte.^175^ The results demonstrated that the “nanopetal”‐structured copper oxide exhibited a *C*_sp_ of 133 F g^−1^ in 0.2 m IL. The deposition of nanostructured copper oxide thin films on stainless steel was achieved through hydrothermal synthesis by Navathe et al.^176^ CV and charge–discharge curves were employed to evaluate the supercapacitance of the thin film electrodes in a three electrode system in a 3 m [HOPMIm][Cl] IL. A *C*_sp_ of 60 F g^−1^ at 0.1 mA cm^−2^ and an ED of 1.18 Wh kg^−1^ was observed at a scan rate of 10 mV s^−1^.

**Figure 5:**
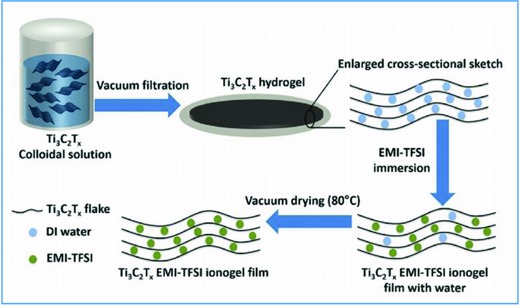
Fabrication of the Ti3C2Tx EMI‐TFSI ionogel film. Reproduced with permission.^177^ Copyright 2016, Elsevier.

An original approach has been followed by Lin et al.^177^ They used an ionogel film of Ti_3_C_2_T*_x_* MXene as electrode, immersed in [EMIm][Tf_2_N] as electrolyte. They explained that this structure allows large sized cations and anions of the IL to penetrate the electrode (**Figure** **5**). The *C*_sp_ was 70 F g^−1^ in a 3 V voltage range, at 20 mV s ^−1^.

Paravannoor et al.^178^ reported highly porous NiO nanowires electrodes coupled with aprotic IL([EMIm][DCA]). Their SCs showed an ED of 21 Wh kg^−1^. Shen et al.^179^ reported [EMIm][Tf_2_N] used in a gel‐based flexible asymmetric SC by electrodepositing g‐FeOOH on carbon electrodes. Pseudocapacitance was attributed to the insertion of IL cations in the material through a diffusion‐controlled process. It displayed its best electrochemical performance at elevated temperatures, i.e., 200 °C, at which 1.44 mWh cm^−3^ of volumetric ED was achieved. A flexible sandwich‐structured SC electrode material has been reported by Sun et al.,^180^ who improved the SC properties by modifying MnO_2_ electrodes with graphene paper itself functionalized by an IL. Graphene was first modified with an NH_2_‐terminated IL [APMIm][Br] to form a freestanding IL–GP, then further modified a MnO_2_ nanosheet by electrodeposition. They reported a *C*_sp_ of 411 F g^−1^ at 1 A g^−1^, of which 70 % is retained after 1000 cycle.

NiO films have appeared as interesting building blocks with diverse functionalities for potential applications in various technologies, not only SCs but also such in LiBs (lithium ion batteries), gas sensors, etc.^181^, ^182^ Using [BMPyr][TFSI] as electrolyte, it was shown that thin films of NiO could be electrodeposited with no intermediate formation of metal hydroxide. Chen et al.^183^ have relied on an hydrothermal method for the synthesis of hierarchical film of b‐Ni(OH)_2_ nanosheets in [BMIm][Cl] which not only helped in the formation of the nanosheets but also helped in promoting growth of the nanosheets on Ni foam substrates. The resulting SCs showed a *C*_sp_ of 700 F g^−1^ at 5 A g^−1^ and long cycle life.

It was found that SnO_2_ nanowires synthesis via chemical vapor deposition showed conductivity ranging from 5 to 70 S cm^−1^ while hypothermally fabricated nanowires of MnO_2_ showed conductivity of about 9 × 10^−4^ S cm^−1^ solution‐based synthesized nanorods of MnO_2_ displayed conductivity of 4 × 10^−2^ S cm^−1^ and bulk MnO_2_ a conductivity of ≈8 × 10^−2^ S cm^−1^. This indicates that the SnO_2_ conductivity is two orders of magnitude higher than that of MnO_2_.

To give a perspective, Yan et al.^184^ reported an easy technique to deposit amorphous MnO_2_ on SnO_2_ nanowires themselves grown on stainless steel (SS), resulting in a SnO_2_/MnO_2_ core/shell nanostructure. Such a strategy proposed some advantages like a) a thin MnO_2_ layer would assist a quick, reversible faradic process and deliver a short ion diffusion route; b) high conductivity SnO_2_ nanowires would offer a straight path for the transport of electrons; and c) SnO_2_ nanowires would allow an efficient diffusion of the electrolyte. The SnO_2_/MnO_2_ composite showed a *C*_sp_ of 637 F g^−1^ at 2 mV s^−1^ of scan rate, in 1 m Na_2_SO_4_ solutions with only a decrease of *C*_sp_ of 1.2% after 2000 CV cycles while a *C*_sp_ of 800 F g^−1^ was obtained through constant current (1 A g^−1^) charge/discharge cycling. These results indicated that the SnO_2_/MnO_2_ composite is an excellent electrode material for SCs and holds great promise if the aqueous electrolyte could be substituted with an IL to increase the potential window.

It is also of interest to underline that organic materials were also reported instead of metal oxide electrodes. For example, finely tuned mesoporous carbon aerogels were used for electrochemical capacitors by Sun et al.,^185^ by employing sol–gel polycondensation of formaldehyde and resorcinol (*m*‐dihydroxybenzene). They reported a *C*_sp_ of 188 F g^−1^ at 1 A g^−1^, a specific energy of 9.1 Wh kg^−1^ at a specific power of 6250 W kg^−1^, and an excellent cyclability.

## 6 Redox ILs

### 6.1 Redox ILs

**Figure 6:**
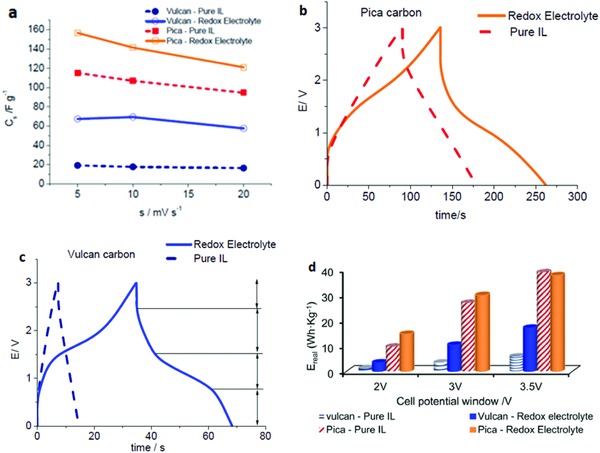
a) Capacitance versus scan rate for SCs with Pica and Vulcan electrodes, for pure PYR14TFSI and for 0.4 m p‐BQ + PYR14TFSI electrolytes at *T* = 60 °C. b) Voltage profiles for Pica and c) Vulcan carbon up to 3 V at 60 °C (constant current density of 10 mA cm^−2^. d) Real energy density (*E*_real_) versus operating voltage of the SCs obtained from charge/discharge cycling at 5 mA cm^−2^. Reproduced with permission.^54^ Copyright 2016, Elsevier.

This exciting new family of ILs bridges redox characteristics with the classical features of the compounds which bring about improvement in charge and energy storage capacity of SCs.^186^, ^187^ With this intent, Navalpotro et al. used redox‐active electrolyte comprising of N‐butyl‐N‐methylpyrrolidinium bis(‐trifluoromethanesulfonyl) imide (PYR14TFSI) and para‐benzoquinone (p‐BQ) composite in an hybrid SC.^54^ The specific capacitance at Pica activated carbon (120–90 F g^−1^) was significantly higher than that of Vulcan activated carbon (15–20 F g^−1^) electrode in pure PYR14TFSI (**Figure** **6**a). The research team reported significant increases in capacitance at Vulcan and Pica activated carbon electrodes, with respectively 70 and 156 F g^−1^, due to addition of electroactive p‐BQ to the IL electrolyte. Furthermore, incorporation of p‐BQ in PYR14TFSI increased discharge time as evidenced by distortion in charge/discharge profile (Figure 6b,c). Likewise, the specific energy in the composite electrolyte increased at three operating voltages (2, 3, and 3.5 V) compared to pure PYR14TFSI electrolyte (Figure 6d). Similarly, Xie et al. used ferrocene‐modified imidazolium cation or bis(trifluoromethanesulfonyl)imide anion as electrolyte in SCs.^188^ The authors noticed a considerable increase (≈80%) in energy density (13.2 Wh kg^−1^) compared to nonfunctionalized IL. Moreover, modification of IL anion with ferrocene suppressed the self‐discharge process owing to thin film deposition at the electrode surface. A similar study conducted by Frackowiak et al. in redox active electrolyte made of potassium iodide and vanadyl sulfate aqueous solutions demonstrated considerable enhancement in capacitance, energy, and power density compared to pristine IL‐electrolyte.^189^ Yamazaki et al. reported high discharge capacitance, good cyclability, and high retention (98%) values in electroactive composite electrolyte made of bromide ions and nonaqueous IL as electrolyte.^190^ Although, introduction of redox active molecules to IL causes considerable increment in capacitance, discharge time, and ED, but still some problems exist which need to be solved, such as, the nature of contribution of redox species toward enhancement of performance and charge storage mechanism in redox active IL‐electrolytes. Moreover, establishment of precise correlation between characteristics of redox electrolytes and porous features of the electrode may also prove vital for progress in this domain.

### 6.2 Biredox ILs

**Figure 7:**
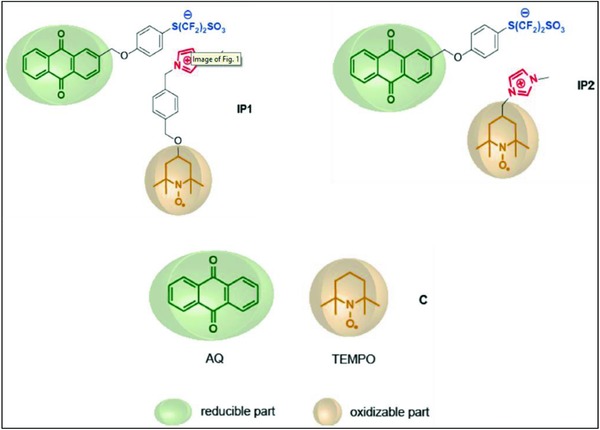
The different types of biredox ionic liquids investigated by Mourad et al. Ion pair 1 (IP1): 2‐methyloxaphenylperflurosulfonate‐anthraquinone and methylimidazolium‐p‐xylyloxa‐TEMPO; IP2: 2‐methyloxaphenylperflurosulfonate‐anthraquinone and methylimidazolium‐TEMPO. Compounds C: anthraquinone AQ and TEMPO. Reproduced with permission.^186^ Copyright 2016, Elsevier.

Biredox‐ILs, a subclass of redox‐IL, are regarded as alternative electrolytes in electrochemical devices including SCs with the objective to enhance electrochemical performances.^191^ Binding a redox group to an ion leads to an enhanced density in liquid phase, approaching the bulk density of redox active solids. By doing so, high capacity and fast redox kinetics like that of bulk redox materials can be achieved. Using the same approach, Mourad et al. used a biredox‐IL in SCs bearing anions and cation functionalized with 2,2,6,6‐tetramethylpiperidinyl‐1‐oxyl (TEMPO) and anthraquinone (AQ) moieties, respectively.^191^ While maintaining cyclic and power stability, the research team documented a doubled increase in specific energy compared to the same cell without electroactive electrolyte. Likewise, Bodin et al. functionalized the cations and anions of bi redox‐IL with AQ and TEMPO groups.^192^ The redox center of BIL underwent fast faradic reaction at positive and negative electrodes compared to other devices utilizing redox species immobilized to the surface. Mourad et al. investigated bioredox‐ILs named IP1 and IP2 comprising of TEMPO tethered to an N‐methylimidazolium cation and AQ tethered to a tetrafluoroethansulfonate anion (**Figure** **7**).^186^ The authors showed that the radius of the electroactive units is the driving factor for electron transfer efficiency.

## 7 Conclusions and Future Outlook

To use ILs as electrolyte in SCs, a systematic and critical study of their physical characteristics is necessary to obtain high capacitances and stabilities. This review demonstrated that, used with ILs, nanoporous carbon‐based electrodes give improved specific capacitance and energy density, either for symmetric or asymmetric SCs. Different promising pseudocapacitors have also been reviewed, using metal oxide‐modified electrodes and bulky ions of selected ILs. Improvements in power and energy density can be obtained through the synergistic coupling of electroactive pseudocapacitive materials and EDLC materials, in aprotic ILs as electrolyte.

Due to their unique physicochemical properties, ILs improve energy and power densities of ECs without compromising on safety and cyclability. However, they are not considered completely benign in terms of toxicity and degradability but seem a plausible choice in comparison to other standard solvents given their high ionic conductivity, nonvolatility, low flammability, good thermal stability, and wide electrochemical stability window, which allows voltage stability and high capacitance.

The most common class of ILs that have been considered so far as prospective electrolytes for SCs are aprotic pyrrolidinium‐ and imidazolium‐based, or containing quaternary ammonium where the cation size plays a significant role in terms of electrolyte viscosity, closely connected to its conductivity. The nature of the cation as well as the length of the alkyl chain has an important effect on the double layer capacitance, which has a direct effect on the electrochemical stability window. It has also been shown that the interfacial contact is affected by the choice of the anion, even if the effect of the anions nature in the IL has not been sufficiently explored and deserves further investigations. One of the key challenges in the field of applications of ionic liquid is to fabricate ILs which exhibit low viscosity and thus increased ionic conductance over a broad range of operational temperatures, i.e., between −30 and +60 °C while demonstrating high thermal stability, with special emphasis on safety for energy storage devices. In many cases, such features are not possible to accomplish in a pure IL. One of the extensively used method to decrease the viscosity and reduce contributions of different sources toward resistance is to use molecular additives that assist in developing strong interactions or miscibility with ionic liquids, in mixtures of ILs and organic solvent. It is worth mentioning that the electrode/electrolyte interface is directly affected by IL's polarizability and, thus, the capacitance. Furthermore, the complete exploitation of EDLC and pseudocapacitive devices based on IL entails additional comprehensive understanding of the mechanisms involved in ion transfer in bulk electrolyte as well as those taking place at the electrode/electrolyte interface. Only a few recent reports really demonstrated the ability of ILs to substitute standard aqueous solvents.

Protic and aprotic ILs, two sub‐classes of ILs, have been extensively studied. The presence of a labile proton on PILs, which has only come lately on the scene, renders them interesting candidates for pseudocapacitors in comparison to their aprotic counterparts. On the contrary, AILs have been widely studied for use in EDLC but are plagued with the issue of using high temperatures to overcome high viscosity. It is being discussed that IL‐based deep eutectic solvents, which rely on cheap raw sustainable materials, are emerging as new electrolytes for electrochemical supercapacitor applications. Hybrid supercapacitors also deserve more investigations. An increase in power and energy densities is achieved through the synergistic coupling of both electroactive pseudocapacitive materials coated with EDLC materials in AIL electrolyte.

However, due to the limited “greenness” of some ionic liquids, it is desirable to focus researches on the synthesis of ILs which do not undergo hydrolysis, do not liberate HCl or hydrofluoric acid (HF) by combustion, and have thermal decomposition temperature above 250 °C, in addition to be biodegradable and require available and cheap raw materials for their preparation. Another important issue should be also kept in mind: although ILs are exciting and may have great potential applications in supercapacitors, they still need to compete with traditional electrolytes in terms of cost.

## Author Contributions

The manuscript was written through contributions of all authors. All authors have given approval to the final version of the manuscript.

## Conflict of Interest

The authors declare no conflict of interest.
